# CTGF Attenuates Tendon-Derived Stem/Progenitor Cell Aging

**DOI:** 10.1155/2019/6257537

**Published:** 2019-11-11

**Authors:** Yun-feng Rui, Min-hao Chen, Ying-juan Li, Long-fei Xiao, Peng Geng, Pei Wang, Zheng-yuan Xu, Xuan-pu Zhang, Guang-chun Dai

**Affiliations:** ^1^Department of Orthopaedics, Zhongda Hospital, School of Medicine, Southeast University, Nanjing, China; ^2^Orthopaedic Trauma Institute, Southeast University, Nanjing, China; ^3^Trauma Center, Zhongda Hospital, School of Medicine, Southeast University, Nanjing, China; ^4^School of Medicine, Southeast University, Nanjing, China; ^5^China Orthopedic Regenerative Medicine Group, Hangzhou, Zhejiang 310000, China; ^6^Department of Geriatrics, Zhongda Hospital, School of Medicine, Southeast University, Nanjing, China

## Abstract

Aged tendon-derived stem/progenitor cells (TSPCs) lead to age-related tendon disorders and impair tendon healing. However, the underlying molecular mechanisms of TSPC aging remain largely unknown. Here, we investigated the role of connective tissue growth factor (CTGF) in TSPC aging. CTGF protein and mRNA levels were markedly decreased in the aged TSPCs. Moreover, recombinant CTGF attenuates TSPC aging and restores the age-associated reduction of self-renewal and differentiation of TSPCs. In addition, cell cycle distribution of aged TSPCs was arrested in the G1/S phase while recombinant CTGF treatment promoted G1/S transition. Recombinant CTGF also rescued decreased levels of cyclin D1 and CDK4 and reduced p27^kip1^ expression in aged TSPCs. Our results demonstrated that CTGF plays a vital role in TSPC aging and might be a potential target for molecular therapy of age-related tendon disorders.

## 1. Introduction

Age-related tendon disorder is one of the main causes of chronic pain, limited joint mobility, and tendon rapture among elderly patients [1, 2]. In tendons, aging reduces the number of tendon cells and decreases their activity [3, 4], thereby depleting the resources required to repair injured tendons. Existing treatments often fail to restore the normal structures and functions of injured tendons [5]. In general, tenocytes were considered to be the only cell type in tendons, which are resident fibroblast-like cells that maintain tendon integrity, remodeling, and repair [4, 6]. Recently, a small population of cells residing in tendons has been identified as stem/progenitor cells exhibiting clonogenicity, self-renewal capacity, and multipotency [7–9]; these stem cells isolated from tendon tissues were termed as tendon-derived stem/progenitor cells (TSPCs). TSPCs could express classical stem cell markers, while maintaining the expression of typical tendon-lineage genes, such as scleraxis (SCX) and tenomodulin (TNMD) [10, 11]. Previous studies suggested that TSPCs could promote tendon repair and regeneration and maintain tendon homeostasis [12, 13]. However, TSPC features alter with advancing age; aged TSPCs display profound self-renewal and differentiation deficit accompanied with premature entry into senescence, which may lead to age-related tendon disorders and impair tendon regeneration [11, 14–16]. So far, the underlying molecular and cellular mechanisms of TSPC aging remain unclear.

CTGF is a cysteine-rich secretory protein belonging to the CCN family and widely expressed in various tissues and organs. CTGF has been implicated as a key regulatory factor in many biological and pathological events including cell adhesion [17], proliferation [18], migration [19], and extracellular matrix (ECM) production [20]. Recent studies have suggested that CTGF is also involved in the regulation of adult stem cells. Lee et al. reported a potent profibrogenic function of CTGF that induces fibrogenic differentiation of MSCs and soft tissue healing in vivo [21]. Yuda et al. reported that CTGF promotes osteo/cementoblastic and fibroblastic differentiation of the human periodontal ligament stem/progenitor cell line [22]. Ni et al. produced an engineered scaffold-free tendon tissue via TSPCs by treatment with CTGF and ascorbic acid in vitro and demonstrated its potentials for neotendon formation and promoting tendon healing in vivo [23]. Istvánffy et al. reported that CTGF maintains cell cycle progression and repopulation activity of hematopoietic stem cells in vitro [24]. Although previous studies have examined the important role of CTGF in stem cells, its role in TSPC aging is still unknown.

In this study, we aim to investigate the CTGF expression pattern of TSPCs in vitro through comparing TSPCs derived from Achilles tendon biopsies of young and aged rats and to examine whether the CTGF could attenuate their aging phenotype. The findings of this study might provide a new molecular target for antagonizing tendon aging.

## 2. Materials and Methods

### 2.1. TSPC Isolation and Culture

The procedures for the isolation of TSPCs from the rat Achilles tendon have been well established [9, 25]. Briefly, rat TSPCs were isolated from 4-month-old (abbreviated as Y-TSPC) and 8-month-old and 20-month-old (abbreviated as A-TSPC) male Sprague-Dawley rats (*n* = 10). The Achilles tendons were gently minced, digested with type I collagenase (3 mg/mL, Sigma-Aldrich), and passed through a 70 *μ*m cell strainer (Becton Dickinson) to yield a single-cell suspension. The released cells were washed in PBS and resuspended in Dulbecco's modified essential medium (DMEM) containing 10% fetal bovine serum, 100 U/mL penicillin, and 100 mg/mL streptomycin (all from Invitrogen). The isolated nucleated cells were plated at an optimal low cell density (50 nucleated cells/cm^2^) for the isolation of stem cells and cultured at 37°C and 5% CO_2_ to form colonies. At day 7, they were trypsinized and mixed together as passage 0 (P0). Cells from P2 to P5 were used for all experiments. Medium was changed every 3 days. The clonogenicity and multilineage differentiation potential of these cells were confirmed before being used for the experiments in this study using standard assays as described previously [9]. All surgical interventions and postoperative animal care were carried out in accordance with the Guide for the Care and Use of Laboratory Animals (National Research Council) and were approved by the Animal Research Ethics Committee of Southeast University. All efforts were made to minimize the number of animals used and their suffering.

### 2.2. Western Blot

After being cultured with or without recombinant CTGF (Cloud-Clone Corp, USA)/CTGF-siRNA, the TSPCs were lysed and centrifuged and the supernatant was then collected for the measurement of protein concentration by the BCA protein assay (Thermo Scientific). 30 *μ*g of protein was denatured, fractionated by electrophoresis on SDS-PAGE, and electrophoretically transferred to a PVDF membrane (Millipore, USA). The blots were blocked with 5% nonfat dry milk in PBST solution and incubated with primary antibodies against CTGF (Proteintech, USA), p16^INK4A^ (Novus Biologicals, USA), p27^KIP1^ (Abcam, USA), CDK4 (Proteintech, USA), cyclin D1 (Bioworld, USA), and *β*-actin (Proteintech, USA) at 4°C overnight. After incubating with a secondary antibody, immunoreactive bands were detected by ECL reagents (Pierce, USA). The gray value of each band was measured, and data are presented as a ratio to *β*-actin.

### 2.3. Immunofluorescence Staining

For immunofluorescence staining, cultured TSPCs were fixed in 4% paraformaldehyde for 15 minutes at room temperature. Cells were blocked with 10% normal serum blocking solution (3% BSA and 0.1% Triton X-100 and 0.05% Tween-20) for 2 h at room temperature. After being washed, cells were incubated overnight at 4°C with anti-CTGF (Proteintech, USA), followed by incubation with a mixture of Alexa Fluor 594-conjugated secondary antibodies (Molecular Probes, USA) for 2 h at room temperature. Immunofluorescence was visualized with a fluorescence microscope (Leica, Germany).

### 2.4. siRNA Transfection

siRNAs were purchased from GenePharma, and cell transfection was performed by CTGF-siRNA when the cells reached 50% confluence. Transfection was performed using Lipofectamine 2000 (Invitrogen, USA). TSPCs were transfected for 72 h, and the transfection mixture was replaced with culture medium.

### 2.5. *β*-Galactosidase Staining

To study the effect of CTGF on cell senescence, the *β*-galactosidase (*β*-gal) assay was performed using the SA-*β*-gal staining kit (Sigma, USA). TSPCs were grown in 12-well plates for 24 h; then, cells were cultured with or without 250 ng/mL CTGF. Cells were incubated with the kit's staining mixture for 16 h at 37°C. The percentages of *β*-gal-positive cells were calculated by counting 300 cells in six microscopic fields.

### 2.6. CCK-8 Assay

The Cell Counting Kit-8 (CCK-8) assay was used to measure cell proliferation. Cells were plated into 96-well culture plates at an optimal density of 3000 cells/well in 200 *μ*L complete culture medium. After 24-hour culture, cells were cultured with or without 250 ng/mL rCTGF. The cells were observed under a microscope, and the CCK-8 assay was performed at 0 h, 24 h, 48 h, and 72 h. 10 *μ*L CCK-8 solution was added to each well and incubated for 2 h at 37°C, and the absorbance of each well was read by the microplate reader at 450 nm.

### 2.7. Microarray Analysis

Gene expression profiles were examined by Beijing CapitalBio Corporation (Beijing, China). Briefly, the poly-A-containing mRNA molecules were purified from the 3 *μ*g of total RNA by using poly-T oligo-attached magnetic beads. The cleaved RNA fragments were reversely transcribed into first-strand cDNA using random hexamers, followed by second-strand cDNA synthesis using DNA polymerase I and RNase H. The cDNA fragments were purified, end blunted, ‘A' tailed, and adaptor ligated. PCR was used to selectively enrich those DNA fragments that have adapter molecules on both ends and to amplify the amount of DNA in the library. The number of PCR cycles was minimized to avoid skewing the representation of the library. The library was qualified by Agilent 2100 Bioanalyzer and quantified by Qubit and qPCR. The produced libraries were sequenced on the HiSeq 2500 platform. After robust multiarray average normalization, a fold change threshold ≥ 2 and *P* value < 0.05 were recognized to be statistically significant alterations. Clustering analysis and heat map generation were performed using Cluster 3.0 software. The functional assignments were mapped onto Gene Ontology (GO).

### 2.8. Quantitative RT-PCR

qRT-PCR was performed as previously described [26]. After being cultured with or without rCTGF/CTGF-siRNA, cells were harvested and homogenized for RNA extraction with the RNeasy Mini Kit (Qiagen GmbH, Germany). The mRNA was reverse transcribed to cDNA by the First-Strand cDNA Kit (Promega, USA). 1 *μ*L of total cDNA of each sample was amplified in the final volume of 20 *μ*L of reaction mixture containing Power SYBR Green PCR Master Mix (Invitrogen, USA) and specific primers using the ABI StepOnePlus system (all from Applied Biosystems). Cycling conditions were denaturation at 95°C for 10 min and 45 cycles at 95°C for 20 s, at optimal annealing temperature for 20 s, at 72°C for 30 s, and finally at 60°C-95°C with a heating rate of 0.1°C/s. The expression of the target gene was normalized to that of the *β*-actin gene. Relative gene expression was calculated as fold change over control and calculated as 2^−ΔΔCt^. The primer sequences used in this study are listed in Supplementary [Supplementary-material supplementary-material-1].

### 2.9. Cell Cycle Analysis

TSPCs were cultured on 10 cm dishes with or without CTGF (250 ng/mL) in 2% FBS/DMEM for 48 h. Then, cells were trypsinized and detached, washed with PBS, and then fixed in 70% ethanol overnight at 4°C. Then, the cells were washed with PBS and incubated with RNase (KeyGen Biotech, China) and propidium iodine (KeyGen Biotech, China) for 30 min. The percentage of cells in the three phases of the growth cycle (G1, S, and G2/M phases) was measured by flow cytometry (Becton Dickinson) using CellQuest software.

### 2.10. Colony-Forming Ability (CFA) Assays

For the CFA assay, 100 cells/cm^2^ of TSPCs were plated in 6-well plates for 10 days in complete media. The cells were stained with 0.5% crystal violet for counting the number of cell colonies. CFA efficiency was estimated as a percentage of counted colonies to the number of plated cells [14].

### 2.11. Population Doubling Time (PDT) Assay

The population doubling time (PDT) assay was performed as described previously [4]. PDT was calculated from the formula log2 [Nc/N0], where N0 refers to the total cell number during seeding and Nc is the total cell number at confluence.

### 2.12. Statistical Analysis

All data are plotted as mean ± standard deviation (SD). Differences in mean values between groups were tested using one-way analysis of variance (ANOVA) followed by Tukey's post hoc multiple comparison test. *P* < 0.05 was considered statistically significant. Each experiment consisted of at least three replicates per condition.

## 3. Results

### 3.1. Expression of CTGF in TSPC Aging

**Figure 1:**
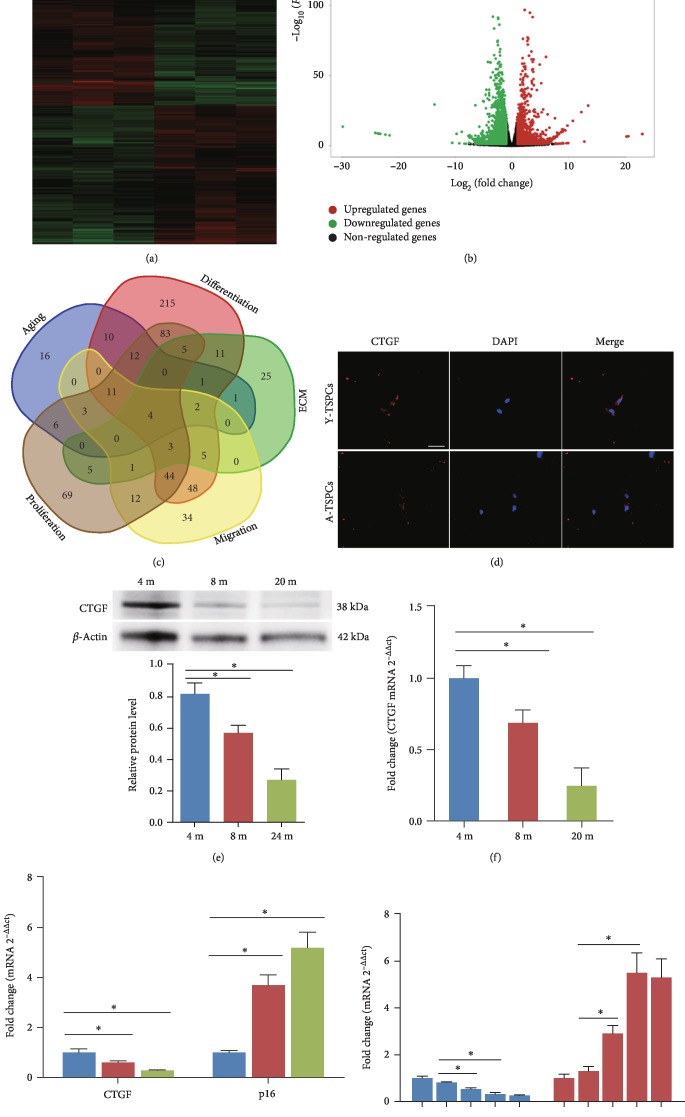
Expression of CTGF in TSPCs. (a, b) Heat map and volcano plot showed genes that were differentially expressed between young and old TSPCs. (c) Venn diagram suggested that CTGF is associated with aging, cell differentiation, migration, ECM, and proliferation in TSPCs. (d) Immunofluorescence staining for CTGF in young and aged TSPCs. (e) Protein levels of CTGF are evaluated by western blotting in TSPCs obtained from 4-, 8-, and 20-month-old rats. (f) CTGF mRNA levels were examined by qRT-PCR. (g) Inverse expressions of CTGF and p16^INK4A^ were assessed by qRT-PCR. (h) Inverse correlation of CTGF and p16^INK4A^ expression levels during expansion and passaging of TSPCs. Scale bars: 20 *μ*m. Values represent the mean ± SD. ^∗^*P* < 0.05, significantly different from the young group.

In the present study, we first examined the mRNA expression profiles with mRNA microarray on young and aged TSPCs. We found 4597 dysregulated mRNAs in aged TSPCs (Figures 1(a) and 1(b)). We then compiled all the predicted genes associated with aging, cell differentiation, migration, ECM, and proliferation for Venn analysis (Figure 1(c) and Supplementary [Supplementary-material supplementary-material-1]), and we found four genes that were related to these functional activities. Among these genes, CTGF has shown a great therapeutic potential in tendon injury in various studies [8, 23, 27]. We then performed immunofluorescent staining to identify the distribution of CTGF in TSPCs. Notably, CTGF was predominantly expressed in the cytoplasm in young TSPCs (Figure 1(d)). However, this cytoplasmic staining was relatively decreased in aged TSPCs. We investigated the expression patterns of CTGF during their progression from young rats (4 months) to aged rats (8 to 20 months). Compared with young TSPCs, CTGF mRNA and protein levels were markedly decreased in the aged TSPCs (Figures 1(e) and 1(f)). Meanwhile, CTGF mRNA levels inversely correlated with those of the senescence marker p16^INK4A^ (Figure 1(g)). This inverse correlation was recapitulated in cultures of TSPCs expanded for 9 passages (Figure 1(h)). The results suggested that CTGF might be associated with TSPC aging.

### 3.2. CTGF Regulates TSPC Aging

**Figure 2:**
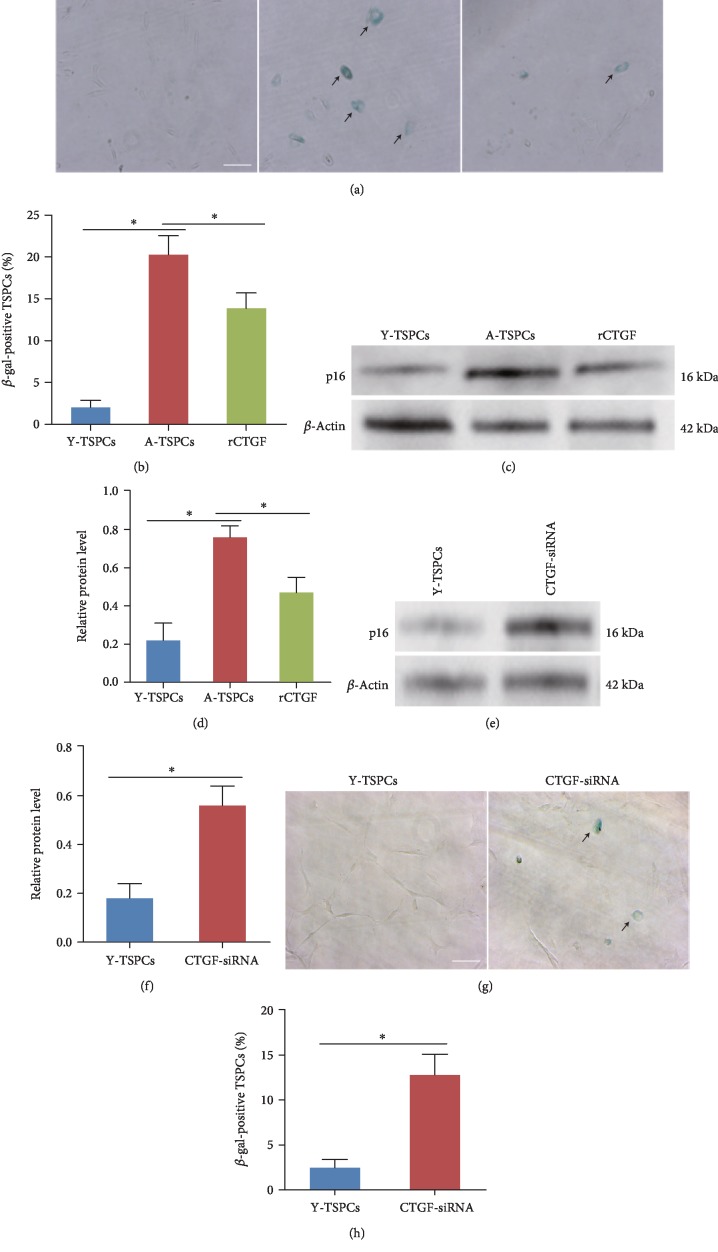
Effects of CTGF on TSPC aging. (a, b) *β*-gal staining showed that rCTGF reduced the number of *β*-gal-positive cells in aged TSPCs. (c, d) Western blotting showed that rCTGF reduced p16^INK4A^ protein level in aged TSPCs. (e, f) CTGF knockdown increased the expression of p16^INK4A^ in young TSPCs. (g, h) *β*-gal staining for the senescent cells in TSPCs. Scale bars: 100 *μ*m. Values represent the mean ± SD. ^∗^*P* < 0.05, significantly different from the young or aged group.

To explore the role of CTGF in TSPC aging, aged cells were treated with recombinant CTGF (rCTGF). *β*-gal staining showed that *β*-gal-positive cells were increased in aged TSPCs (20 months), and this increment was rescued by the addition of rCTGF (Figures 2(a) and 2(b)), supporting its senescence-delaying potential. In addition, western blot showed that p16^INK4A^ expression was decreased after rCTGF treatment (Figures 2(c) and 2(d)). Then, CTGF-siRNA was used to knockdown CTGF in young TSPCs. The result showed that CTGF deletion increased the expression of p16^INK4A^ in young TSPCs (Figures 2(e) and 2(f)). Moreover, *β*-gal staining showed that CTGF deletion also increased the percentage of *β*-gal-positive cells in young TSPCs (Figures 2(g) and 2(h)). All these results suggest the specific role of CTGF in TSPC aging.

### 3.3. CTGF Positively Affects Aged TSPC Self-Renewal

**Figure 3:**
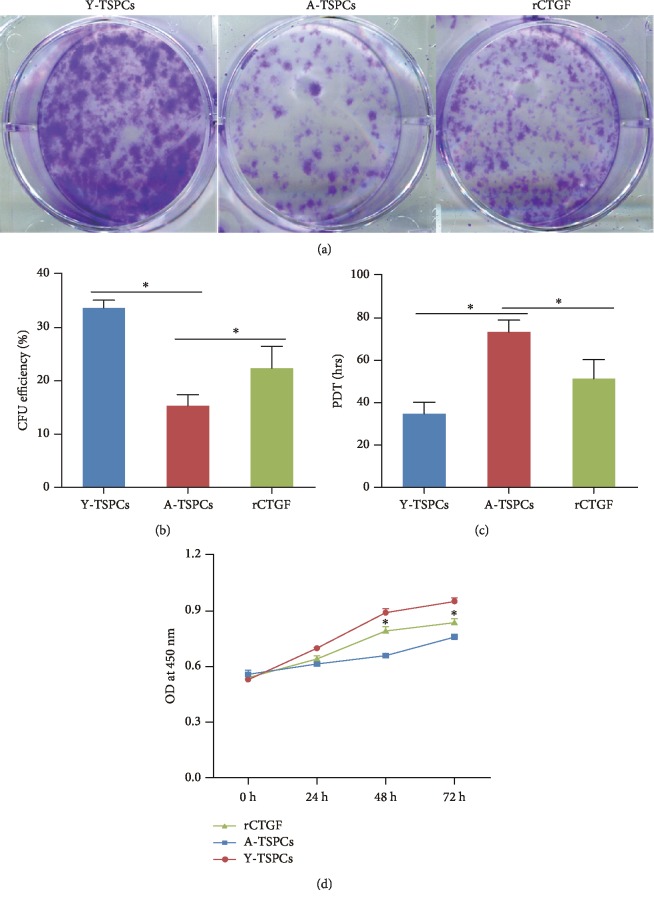
Effects of CTGF on self-renewal of TSPCs. (a) Colony-forming unit assays. Colonies were stained with crystal violet at day 14. (b) Proliferation of young, aged, and rCTGF-treated aged TSPCs in culture measured by the PDT assay. (c) CCK-8 assay showed that rCTGF significantly increased the proliferative rate of aged TSPCs. Values represent the mean ± SD. ^∗^*P* < 0.05, significantly different from the young or aged group.

To investigate self-renewability of TSPCs, we performed colony-forming ability (CFA) assays (Figures 3(a) and 3(b)), population doubling time (PDT) assay (Figure 3(c)), and CCK-8 assay (Figure 3(d)). We found a slower clonogenic ability and proliferation rate in aged TSPCs when compared with those in young TSPCs. Notably, rCTGF treatment significantly increased the colony number and proliferative rate compared with aged TSPCs.

### 3.4. Recombinant CTGF Increased the Tendon-Related Marker Expressions of Aged TSPCs

**Figure 4:**
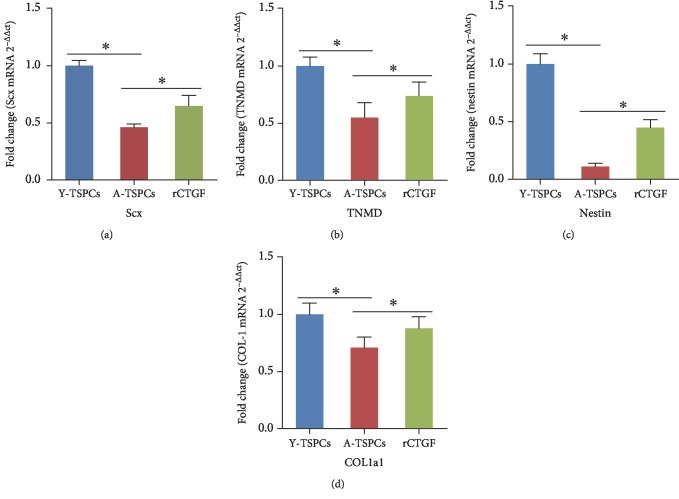
CTGF increased the tendon-related gene expression. (a–d) qRT-PCR analysis of Scx, Tnmd, nestin, and Col1a1 transcript levels in young, aged, and rCTGF-treated aged TSPCs. Values represent the mean ± SD. ^∗^*P* < 0.05, significantly different from the young or aged group.

qRT-PCR was performed to evaluate the key tendon-related markers of TSPCs treated with rCTGF. Compared with young TSPCs, the mRNA levels of Scx, Tnmd, nestin, and Col1a1 were reduced in aged TSPCs (Figures 4(a)–4(d)), which suggested that aging suppresses the key tendon-related marker expression of TSPCs. In addition, rCTGF significantly increased these gene expressions in aged TSPCs.

### 3.5. CTGF Regulates TSPC Aging through Cell Cycle Progression

**Figure 5:**
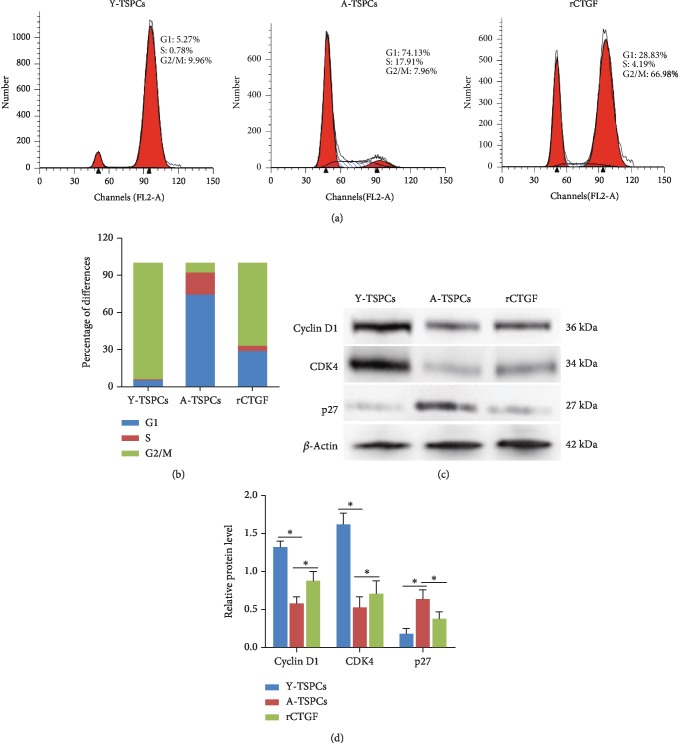
The effect of CTGF on the cell cycle in TSPCs. (a, b) The cell cycle distribution of TSPCs was detected by flow cytometry. (c, d) Expression of cyclin D1, CDK4, and p27^kip1^ in TSPCs is evaluated by western blotting. Values represent the mean ± SD. ^∗^*P* < 0.05, significantly different from the young or aged group.

We investigated the cell cycle distribution among TSPCs by flow cytometry. Compared with young TSPCs, the proportion of aged TSPCs was significantly increased in the G1/S phase, indicating cell cycle arrest at the G1/S checkpoint (Figures 5(a) and 5(b)). Furthermore, the accumulation of aged TSPCs at the G1/S phase was blocked after rCTGF treatment (Figures 5(a) and 5(b)). Then, we investigated the expression of the key cell cycle-rated proteins that regulate the G1/S phase transition; the result showed that cyclin D1 and CDK4 expression was decreased, while the expression of p27^kip1^ was increased in aged TSPCs when compared with that in young TSPCs (Figures 5(c) and 5(d)). Moreover, rCTGF treatment notably increased cyclin D1/CDK4 protein levels and reduced p27^kip1^ expression in aged TSPCs when compared with those in young TSPCs.

## 4. Discussion

TSPC aging is one of the major risk factors for tendon degeneration and injury. But the molecular and cellular mechanisms regulating this process are still unclear. In the current study, we demonstrated for the first time that CTGF plays a vital role in TSPC aging. Our study showed that the CTGF expression in TSPCs declines with age. Meanwhile, recombinant CTGF suppresses TSPC aging and promotes self-renewal and tendon-related marker expressions of aged TSPCs. We also found that the cell cycle distribution of aged TSPCs were arrested in the G1/S phase, and recombinant CTGF could restore the arrest.

Previous studies have demonstrated that CTGF regulates the expression of collagens and glycoproteins in tendons and recombinant CTGF treatment could promote healing of the tendon [8, 28], which indicated the important role of CTGF in tendon healing. To date, only a few studies focused on the role of CTGF in tendon aging. In this study, we showed the expression profile of CTGF in aged TSPCs. With advancing age, CTGF was significantly decreased at both protein and mRNA levels. We then investigated its effect on aging, self-renewal, and differentiation of aged TSPCs.

As mentioned above, a study has shown that CTGF prevents cell cycle arrest and senescence of hematopoietic stem cells [24]. In this study, we found similar results in TSPCs. Our results showed that CTGF reduced the number of *β*-gal positive senescent cells, which suggests the ability to prevent TSPC aging. p16^INK4A^ is a cell cycle inhibitor and plays a vital role in stem cell aging [29–31]. Studies have shown that p16^INK4A^ expression is increased in aged TSPCs [32, 33]. Here, we also showed that p16^INK4A^ expression was increased in aged TSPCs and recombinant CTGF could reduce p16^INK4A^ expression. The results suggest the critical role of CTGF in TSPC aging.

TSPCs differ from tenocytes in their ability to self-renew and differentiation potential [34]. Dysfunction of self-renewal and differentiation appears to be a hallmark of TSPC aging [35]. Self-renewal ability determines stem cells as the major cell type responsible for tissue homeostasis and regeneration [32]. In the present study, we investigated TSPCs' self-renewal by performing CFA assays, PDT assay, and CCK-8 assay. The results showed that clone-forming ability and proliferative rate of aged TSPCs were significantly decreased, which indicates self-renewal dysfunction. In addition, recombinant CTGF could increase the colony number and proliferative rate in aged TSPCs, supporting a critical role of CTGF in regulating the ability of self-renewal of aged TSPCs. A previous study has also shown that CTGF promotes the proliferation of rat TSPCs [8], which supports our results.

Consistent with inferior self-renewal ability of aged TSPCs, the expression levels of key tendon-related markers, Scx, TNMD, nestin, and COL1a1, were reduced, which indicated the impaired tenogenic differentiation ability of aged TSPCs. Scx and TNMD are important for tenogenic differentiation in tendon cells [10, 36, 37]. A previous study has shown that nestin-positive TSPCs exhibited superior tenogenic capacity compared to nestin-negative TSPCs [38]. COL-1 is the main extracellular matrix of the tendon and maintains the normal structure [39]. Moreover, recombinant CTGF treatment significantly increased these key tendon-related marker expressions in aged TSPCs, which indicated the positive role in regulating TSPC tenogenic differentiation.

Previous studies suggested the crucial role of CTGF in the cell cycle, especially in the G1/S phase [22, 24]. Here, we investigated the cell cycle distribution among TSPCs; we found that aged TSPCs were arrested in the G1/S phase, while rCTGF treatment promoted G1/S transition. We also investigated expressions of cyclin D1, CDK4, and p27^kip1^ in TSPCs. Cyclin D1 and CDK4 are representative proteins of the G1/S phase, and p27^kip1^ is a key inhibitor of the G1/S phase in mammalian cells [40]. We found that recombinant CTGF treatment notably increased cyclin D1/CDK4 protein levels and reduced p27^kip1^ expression compared with young TSPCs. As the stem cell aging is controlled by cell cycle progression [41, 42], the results suggested that CTGF antagonizes TSPC aging by maintaining the cell cycle progression.

## 5. Conclusions

**Figure 6:**
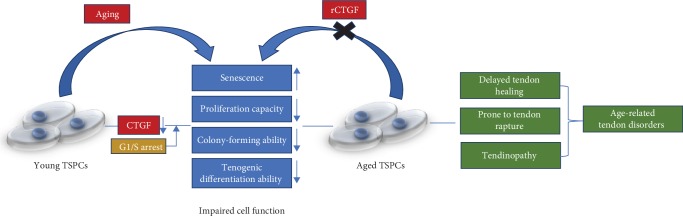
Model by which CTGF regulates TSPC aging. CTGF expression declines with age in TSPCs. In aged TSPCs, reduction of CTGF expression causes cell cycle arrest, leading to impaired cell function.

In summary, we demonstrated that CTGF could restore the age-related decline in repairing capacity of the aged TSPCs with attenuated senescence, increased proliferation capacity, colony-forming ability, and tenogenic differentiation ability (Figure 6). All these findings suggested that CTGF plays a critical role in TSPC aging; these features of CTGF imply that it could be efficient as an optimal therapeutic agent for age-related tendon disorders.

## Data Availability

The data used to support the findings of this study have not been made available because the research work about CTGF in TSPCs is not fully completed. The further study is still in progress.

## Supplementary Materials

Supplementary Materials The supplementary material contains two tables. Table 1 lists primer sequences for qRT-PCR. Table 2 shows the genes associated with aging, cell differentiation, migration, ECM, and proliferation for Venn analysis.
