# Supplementary material for: CTGF Attenuates Tendon-Derived Stem/Progenitor Cell Aging
Source: Stem Cells Int. 2019 Nov 11;2019:6257537. doi: 10.1155/2019/6257537 (PMC6881574; doi:10.1155/2019/6257537)
Supplement: Supplementary Materials — The supplementary material contains two tables. Table 1 lists primer sequences for qRT-PCR. Table 2 shows the genes associated with aging, cell differentiation, migration, ECM, and proliferation for Venn analysis. [file 6257537.f1.docx]

TABLE S1. Primer sequences for qRT-PCR.

| Target gene | Accession No. | Forward | Reverse | Amplicon (bp) |
| --- | --- | --- | --- | --- |
| CTGF | NM_022266.2 | 5′-CCAACTATGATGCGAGCCAACT-3′ | 5′-TTAGCCCGGTAGGTCTTCACACT-3′ | 272 |
| p16^INK4A^ | NM_031550.1 | 5′- CCGATACAGGTGATGATGATGG-3′ | 5′- ACCGCAAATACCGCACGAC-3′ | 253 |
| Nestin | NM_001308239.1 | 5′-TGGAGCAGGAGAAGCAAGGTC-3′ | 5′-CAAGGGGGAAGGGAAGGATGT-3′ | 281 |
| TNMD | NM_022290.1 | 5′- GACCTATGGCATGGAGCACAC-3′ | 5′- TGTTTCATCGGTGCCATTTCC-3′ | 118 |
| Scx | NM_001130508.1 | 5′ - CGAGAACACCCAGCCCAAAC-3′ | 5′- CGTCTTTCTGTCACGGTCTTTG-3 | 82 |
| COL1a1 | NM_053304.1 | 5′-AGAGGCATAAAGGGTCATCGTG-3′ | 5′-AGACCGTTGAGTCCATCTTTGC-3′ | 161 |
| β-actin | NM_031144 | 5′ - TGCTATGTTGCCCTAGACTTCG -3′ | 5′- GTTGGCATAGAGGTCTTTACGG -3′ | 240 |

| Table s2. Biological functions and genes affected in aged TSPCs | |
| --- | --- |
| Categories | Genes |
| Aging | Rgn, Igfbp3, Il6, Tyms, Msh2, Pth1r, Hmga1, Col3a1, Cnr1, Eng, Tgfb2, RGD1305645, Fgf2, Hp, Aurkb, Rpl29, Glrx, Mme, Srebf2, Kat6a, Ggt1, Ypel3, Hmgcr, Calr, Met, Tfrc, Socs2, Ucp2, Ednrb, Ncf2, Aldh3a1, Nr5a1, Ldlr, Pde4d, Fosl2, Dnmt1, Kras, Sod1, Edn1, Agtr1a, Kcnma1, Tfcp2l1, Fads1, Ncam1, Mmp9, Bcl2, Eno3, Foxm1, Prmt6, Acan, Prelp, Tgfbr2, Vcam1, Cx3cl1, Npy, Penk, Ireb2, Gsta2, Ctgf, Vash1, Ntrk1, Kdr, Fsbp, Igfbp2, Top2a, Atr |
| Differention | Nab2, RGD1309676, Clic4, Cobl, Dhx9, Ntng2, Pkdcc, Tgm1, Pth1r, Tjp2, Exoc4, Nfkbia, Enpp2, Ctf1, Foxc2, Soat1, Ddit4, Fgf2, Sema7a, Mdfi, Dpysl4, Axl, Apold1, Ift20, Slc2a4, Pglyrp1, Radil, Axin2, Vnn1, Egr1, Met, Snai1, Slc12a5, Tfrc, Col1a1, Hoxc8, Dcdc2, Snai2, Dkkl1, Xrcc2, Fbn2, Nr5a1, Mafb, Dsp, Hmgb2l1, Tmem204, Uqcrq, F2rl1, Actr2, Sema5a, Emx2, Lpin1, Ptprq, Sod1, Edn1, Kcnma1, Cdc20, Fmod, Egr2, Lrp6, Foxm1, Notch2, Tgfbi, Cd53, Isg15, Gpsm2, Sema4f, Tlr4, Efna4, Itga1, Tgif1, Mstn, Akap6, Tmod2, Flrt2, Rab38, Penk, Trib2, Htr2b, Enpp3, Mt2A, Bnip3, Aspa, Vcl, Sema3a, Plk5, Ctgf, Ogn, Atxn1, Slit1, Selk, Kdr, Megf10, Alg10, Cdkn2c, Lpl, Lfng, Tpm4, Cspg4, Cebpb, Igfbp3, Spp1, Twist2, Rhoh, Mmp15, Lrrc8c, Folr1, Col3a1, Cnr1, Tmem178a, Dhfr, Il1rl2, Col7a1, Ccnd1, Rasgrf1, Frmd6, Pdlim5, Errfi1, Nebl, Sulf2, Epb4.1l5, Isl1, Nefm, Ankrd1, Plagl1, Fam65b, Wwtr1, Rack1, Stac3, Elf1, Ednrb, Duxbl1, Camk2d, Eml1, Plac8, Slc6a4, Ptn, Epha7, Pak3, Il23a, Dab2ip, Inpp4b, Apoa1, Cyp26b1, Fbxo5, Picalm, Unc13a, Pak2, Brinp2, Acvrl1, Prrx1, Ezh2, Lama5, Amer1, Epas1, Robo1, Ncam1, Avil, Mmp9, Iqgap1, Bcl2, Aspm, Erbb4, Tnni3, Ccr1, Spata24, Angptl4, Gdf15, Foxo1, NEWGENE_69363, Sema6b, Mei1, Trim6, Traf6, Il3ra, Runx2, Sema3g, Pde3a, Mmp11, Gnaq, Cmklr1, Itga6, Myh9, Cx3cl1, Mkx, Plxnd1, Btg2, Dclk1, Dlk1, Hes1, Tubb2b, Per2, Insc, Trpm1, Lgr5, Pgr, NEWGENE_1306455, Rpl22, Casp4, Ier2, Ndn, Tet2, Hspa5, Wfdc21, Dmrt3, Gpc2, Rpl24, Top2a, Arhgef26, Plcl2, Tubb3, Lrrc8a, Galntl5, Cdhr5, Ptk2b, Spag9, Il7r, Cd24, Mtr, Tmem8c, Phf19, Uhmk1, Kif26a, Fermt3, Ar, Cyp11a1, Slc9b2, Pacsin1, Rgs20, Mmp13, Kat6a, Insl6, Nes, Strn, Hmgcr, Tmem176b, Bmp3, Hist1h2ao, Calr, Cdc25b, Fam101b, Homer1, Fanca, Lpar3, Socs2, Cebpd, Gsc, Hoxd9, Shank3, Btbd3, Ppp3cb, Zfp521, Cxadr, Fosl2, Mcoln3, Chrdl1, Cit, Rps3a, Sema4a, Rps6ka2, Smarcb1, Kras, Ddias, Aldh1a2, Wisp1, Gdf6, Rap1gap2, Mnda, Tek, Rapgef4, Nrp1, Satb1, Bcl2l11, Unc5a, Tfcp2l1, Lif, Gfra3, Ifrd1, Klhl41, Serpinf2, Col11a1, Lmnb1, Pcp4, Trib3, Fancd2, Nr5a2, Klf7, Fuom, Ss18l1, Gstk1, Musk, Meis1, Dlk2, Hdac11, Mdk, Tgfbr2, Baiap2, Magi2, Stmn2, Mlf1, Kitlg, Rgs2, Tfap2c, Apcdd1, Sfn, Ptprm, Xdh, Nupr1, Ireb2, Epha2, Angpt1, Foxp3, Etv3, Gpc3, Fez1, Fam83d, Rps15, Zpbp2, Flrt3, Dhcr7, Osr2, L1cam, Cib1, Socs1, Ntrk1, RGD1562378, Hoxc11, Nr2f1, Ttl, Zhx3, Trip13, Zbtb7c, Tead2, Adamts9, Il6, Ptpro, Tyms, Ubash3b, Msh2, Ccdc67, Racgap1, Eng, Hmgb2, Tgfb2, Srsf1, Cd109, Pls3, Rsph1, Emp2, Insig1, Rims2, Rimbp3, Wif1, Unc13d, Pi16, Myh9l1, Sprr1a, Mgmt, Rasa1, Cux2, Fli1, Fam20c, Tesmin, Msn, Ebf2, Crmp1, Grin2a, Sema3d, Mtmr2, Ptprg, Sh2b2, Itgb3, Fzd1, Zfp36, Ppp2r2b, Ret, Medag, Mycn, Sall1, Cav2, Adam9, Scd, Map3k1, Duoxa1, Pde4d, Tenm4, Syt4, Dnmt1, Chac1, Mdga2, Gata4, Rps6, Csf3r, Hopx, Cntf, Adcyap1r1, Pax1, Mgll, Hap1, Tmem64, Lilrb3b, Olig1, Rps14, Ell3, Clic5, Pcsk9, Cntn1, Dock1, Ccnb1, Psen2, Tlr2, Wdr62, Kalrn, Acan, Klf1, Fat3, Irx3, Agtpbp1, Sema6c, Ghr, Muc1, Ccl3, Fasn, Angptl8, Rab7b, Inha, Mgst1, Frzb, Ntn3, Fdps, Npy, Csf2, Rps19, Txnip, Fignl1, Aacs, Bambi, Metrn, Lilrb4, Hif1an, Draxin, Fgf9, Psen1, Msi1, Gpld1, Grk5, Ccnd2, Slitrk2, Spata18, Adcy1, Pla2g2a, Sema3c, Col5a1, Igsf9 |
| Extracellular matrix | Omd, Hapln4, Adamts9, Mfap4, Lamc2, Kazald1, Mmp15, Ltbp1, Col3a1, Tgfb2, Cpz, Col7a1, Fcgbp, Lox, Mmp13, Rell2, Calr, Thbs1, Adamts4, Fbln5, Slpi, Col1a1, Fbn2, NEWGENE_1549776, Cask, Ptn, Adamts15, Wisp1, Sod1, Adamts6, Lama5, Tnfrsf11b, Gsto1, Fmod, Mmp9, Col11a1, Nav2, Hsp90b1, Angptl4, P3h2, Acan, Hapln1, Prelp, Tgfbi, Aspn, Mmp11, Itga6, Spon2, Flrt2, Thbs2, Adamts5, Ogn, Ctgf, Col6a2, Fgf9, Gpc3, Gpld1, Slit1, Odam, Wisp2, Gpc2, Col5a1, Lpl |
| Migration | Clic4, Hoxb9, Ptk2b, Spag9, Cd24, Sdc3, Enpp2, Foxc2, Fermt3, Ddit4, Fgf2, Sema7a, Ccl20, Dpep1, Ccdc141, Srgap3, Axl, Grb7, Calr, Egr1, Met, Snai1, Col1a1, Dcdc2, Snai2, Cd274, Cxadr, Hmgb2l1, Sema4a, F2rl1, Sema5a, Emx2, Tek, Rapgef4, Nrp1, Gcnt1, Edn1, Gfra3, Cpne3, Gpsm3, Lrp6, Ccl11, Mdk, Tgfbr2, Sema4f, Cln3, Magi2, Itga1, Mcam, Kitlg, Ccl27, Apcdd1, Flrt2, Ptprm, Htr2b, Pdgfc, Vcl, Epha2, Angpt1, Sema3a, Ctgf, Vash1, Fam83d, Flrt3, Slit1, Grm1, Ddx58, Cib1, Selk, Srgap1, Kdr, Nr2f1, Trem3, Ephb4, Kctd13, Pla2g7, Cspg4, Pde4b, Igfbp3, Spp1, Ptpro, Pstpip2, Col3a1, Folr1, Eng, Tgfb2, Hmgb2, Cxcl2, Emp2, Vav3, Myh9l1, Dapk2, Podxl2, Epb4.1l5, Msn, Isl1, Avl9, Sema3d, Ptprg, Thbs1, Itgb3, Mst1, Rack1, Ednrb, Ret, Camk2d, Adam9, Ptn, Rab5al1, Pak3, Map3k1, Pde4d, Il23a, Dab2ip, Csf3r, Apoa1, Slk, P2ry6, Depdc1b, Acvrl1, Lama5, Prr5l, Robo1, Agtr1a, Tac1, Iqgap1, Mmp9, Aspm, Bcl2, Erbb4, Dock1, Tlr2, Ccr1, Sema6b, Kalrn, Acan, Fat3, Sema3g, Prkce, Sema6c, Ccl3, Itga6, Cmklr1, Vcam1, Cx3cl1, Myh9, Plxnd1, Rps19, Vegfb, Dclk1, Dock5, Bambi, Ch25h, Hes1, Tubb2b, Adra2a, Psen1, Gpld1, Pgr, Zfp580, Pycard, Ndn, Sema3c, Hspa5, Col5a1, C5ar2, Il16 |
| Proliferation | Tgm1, Pth1r, Slc39a10, Ncapg2, Hmga1, Hmox1, Tspyl5, Nfkbia, Cdca7l, Enpp2, Foxc2, Ddit4, Fgf2, Prc1, Ift20, Fth1, Axin2, Egr1, Met, Tfrc, Snai2, Hmgb2l1, Pemt, F2rl1, Sema5a, Emx2, Edn1, Atad5, Tnfrsf11b, Cdc20, RGD1310335, Lrp6, Ptger2, Ccl11, Foxm1, Notch2, Tgfbi, Dusp22, Tlr4, Rrm1, Itga1, Adora2b, Tgif1, Mstn, Penk, Htr2b, Plk5, Ctgf, Rrm2, Pde1a, Ogn, Vash1, Atxn1, Selk, Kdr, Megf10, Cdkn2c, Odam, Igfbp2, Mtbp, Cebpb, Cspg4, Igfbp3, Cdc7, Twist2, Cdca7, Ace2, Esm1, Tgm2, Cxcl2, Ccnd1, Tk1, Rasgrf1, Aurkb, Errfi1, Sulf2, Isl1, Wwtr1, Thbs1, Ednrb, Duxbl1, Camk2d, Eml1, Plac8, Slc6a4, Ptn, Il23a, Dab2ip, Il11, Apoa1, Gins1, Picalm, Cdc6, Acvrl1, Prrx1, Lama5, Ezh2, Las2, Agtr1a, Robo1, Ncam1, Mmp9, Bcl2, Aspm, Erbb4, Spta1, Fmc1, NEWGENE_69363, Foxo1, Traf6, Runx2, Gnaq, Dhcr24, Cx3cl1, Rbp4, Btg2, Pola1, Crlf1, Hes1, Per2, Hoxd13, Zfp580, Lgr5, Pgr, Ndn, Wisp2, C5ar2, Plcl2, Ptk2b, Cd24, Melk, Il7r, Akr1b1, Npr3, Mki67, Ar, Il13ra1, Egln3, Nes, Strn, Hmgcr, Calr, Fanca, Palb2, Avp, Cd274, Cxadr, Cd180, Fosl2, Camp, Cd40, Rps3a, Rps6ka2, Smarcb1, Kras, Aldh1a2, Wisp1, Mnda, Egf, Tek, Satb1, Lif, Ifrd1, Klhl41, Serpinf2, Map3k2, Rps4x, Nr5a2, P3h2, Mab21l1, Tgfbr2, Magi2, Dio3, Kitlg, Tfap2c, Ptprm, Sfn, Xdh, Hmx2, Apln, Nupr1, Epha2, Pdgfc, Angpt1, Hells, Foxp3, Etv3, Gpc3, Fam83d, Dhcr7, Cib1, Osr2, Ntrk1, Mab21l2, Rgn, Alox15, Sat1, Il6, Ogfod1, Racgap1, Eng, Mcm10, Hmgb2, Tgfb2, Cd109, Srpx, Emp2, Vav3, Rpl29, Lrrc32, Rasa1, Itgb3, Mst1, Zfp36, Mycn, Mfn2, Cav2, Sall1, Cask, Aldh3a1, Rab5al1, Pde4d, Tenm4, Dnmt1, Rps6, Gata4, Cntf, Igfbp4, Pax1, Ell3, Cd38, Tac1, Foxp2, Uts2r, Ccnb1, Tlr2, Iqgap3, Rps15a, Kalrn, Ptgir, Vcam1, Angptl8, Frzb, Inha, Npy, Csf2, Vegfb, Fignl1, Txnip, Bambi, Fgf9, Psen1, Grk5, Gpld1, Ccnd2, Pla2g2a, Tcf19, Pycard |
